# Sequencing and Characterization of the Invasive Sycamore Lace Bug *Corythucha ciliata* (Hemiptera: Tingidae) Transcriptome

**DOI:** 10.1371/journal.pone.0160609

**Published:** 2016-08-05

**Authors:** Fengqi Li, Ran Wang, Cheng Qu, Ningning Fu, Chen Luo, Yihua Xu

**Affiliations:** Institute of Plant and Environment Protection, Beijing Academy of Agriculture and Forestry Sciences, Beijing, 100097, P. R. China; Institute of Vegetables and Flowers, Chinese Academy of Agricultural Sciences, CHINA

## Abstract

The sycamore lace bug, *Corythucha ciliata* (Hemiptera: Tingidae), is an invasive forestry pest rapidly expanding in many countries. This pest poses a considerable threat to the urban forestry ecosystem, especially to *Platanus* spp. However, its molecular biology and biochemistry are poorly understood. This study reports the first *C*. *ciliata* transcriptome, encompassing three different life stages (Nymphs, adults female (AF) and adults male (AM)). In total, 26.53 GB of clean data and 60,879 unigenes were obtained from three RNA-seq libraries. These unigenes were annotated and classified by Nr (NCBI non-redundant protein sequences), Nt (NCBI non-redundant nucleotide sequences), Pfam (Protein family), KOG/COG (Clusters of Orthologous Groups of proteins), Swiss-Prot (A manually annotated and reviewed protein sequence database), and KO (KEGG Ortholog database). After all pairwise comparisons between these three different samples, a large number of differentially expressed genes were revealed. The dramatic differences in global gene expression profiles were found between distinct life stages (nymphs and AF, nymphs and AM) and sex difference (AF and AM), with some of the significantly differentially expressed genes (DEGs) being related to metamorphosis, digestion, immune and sex difference. The different express of unigenes were validated through quantitative Real-Time PCR (qRT-PCR) for 16 randomly selected unigenes. In addition, 17,462 potential simple sequence repeat molecular markers were identified in these transcriptome resources. These comprehensive *C*. *ciliata* transcriptomic information can be utilized to promote the development of environmentally friendly methodologies to disrupt the processes of metamorphosis, digestion, immune and sex differences.

## Introduction

The sycamore lace bug, *Corythucha ciliata* (Say, 1832) is a native North American insect that specifically feeds on *Platanus* spp. (Platanaceae), including *P*.× *occidentalis*, *P*. ×*acerifolia* and *P*. ×*orientalis* [1,2]. The bugs feed on the undersides of the leaves of plane trees (*Platanus* spp.), leading to a white stippling that eventually progress into chlorotic or bronzed foliage and premature senescence of leaves [1]. *C*. *ciliata* may transmit two fungi, *Ceratocystis fimbriata* Hell. et Halsted *forma specialis platani* Walter and *Apiognomonia* (= *Gnomonia*) *veneta* (Sacc. and Spreg.)[3]. The insulted trees will defoliate earlier, stop upward growth, become weak and even die [4,5]. A recent study found that *C*. *ciliata* can suck human blood, leading to health problem in human [6].

*C*. *ciliata* was first discovered in Europe in 1964 in Padova, Italy and has now spread through many countries in the world [1,7]. *C*. *ciliata* migrates quickly and now it has been detected in at least 26 cities of 12 provinces since it was first found in 2002[8] in China, including Hunan, Hubei, Shanghai, Zhejiang, Jiangsu, Shandong, Henan, Chongqing and Guizhou, etc [9]. This pest has been included in the list of dangerous forest pest and it was even listed as a moderate dangerous forest pest by the Chinese Forestry Administrative Department in 2007[10].

Currently, the management of *C*. *Ciliata* was depended heavily on the use of insecticides. However, large-scale application of insecticides can lead to many problems in urban area[1]. In addition, *C*. *ciliata* has developed the tolerances to insecticides with phosphorus acid ester ingredient [11]. Faced with this situation, the development of effective and environmentally sound novel management strategies including behavioral manipulations[12], neurophysiological manipulations[13] and RNAi methods[14] was necessary. The genomic and transcriptomic resources of *C*. *ciliata* have been needed to aid the development of novel method for managing this insect. Furthmore, *C*. *ciliata* represents one of a group of important agricultural and forestry pests, highly invasive herbivorous bugs of the Tingidae family-which include avocado lace bug, *Pseudacysta perseae*, subsocial lace bug, *Gargaphia solani*, azalea lace bug *Stephanitis pyrioides* (Scott), and Chrysanthemum lace bug, *Corythucha marmorata* (Uhler,1878)[15–17]. Thus, the *C*. *ciliata* transcriptome could serve as a reference transcriptome for promoting the molecular biology and evolutionary studies of Tingidae species.

In the present study, we sequenced and analyzed the *C*. *Ciliata* transcriptomes from three samples represented difference developmental stages and sex difference. This is the first species of Tingidae where the RNA has been sequenced. The sequenced, assembled and annotated transcriptome datasets will provide assistance with the identification of genes associated with *C*. *Ciliata* metamorphosis, digestion and immune, and sex difference, help to better analyses of the genetic function, reveal molecular regulatory mechanisms and develop molecular marker.

## Materials and Methods

### Insect materials and RNA isolation

*C*. *Ciliata* samples (nymphs, AM and AF) were collected from the *P*.×*acerifolia* trees grown in the garden of plant protection institute, Chinese Academy of Agricultural Science, Haidian District (40°01’24.13”, 116°16’46”) in September 2015. The AF and AM were distinguished based on the abdomen morphology[9]. Nymphs were mix 3^th^ and 4^th^ instar nymphs. Nearly 500 individuals of each sample (nymphs, AF and AM) were used in RNA extraction. Total RNA was isolated using the RNAqueous-Micro kit (Life Technologies), and the integrity of all RNA samples was assessed using Agilent Bioanalyzer 2100 system (Agilent Technologies, CA, USA) with a minimum RNA integration value of 6.

### cDNA library preparation and Illumina sequencing

To obtain the comprehensive gene information of *C*. *Ciliata*, Illumina sequencing was performed for all three *C*. *Ciliata* RNA samples represented the different developmental stages and sex differences. All cDNA libraries were generated in accordance with the manufacturer’s protocol (Illumina Inc., San Diego, CA, USA). 1.5 μg RNA per sample of *C*. *Ciliata* developmental stages was used for library construction. Sequencing libraries were generated using NEBNext^®^ Ultra^™^ RNA Library Prep Kit for Illumina^®^ (NEB, USA) following manufacturer’s recommendations. Briefly, mRNA was purified from total RNA using poly-T oligo-attached magnetic beads. Fragmentation was carried out using divalent cations under elevated temperature in NEBNext First Strand Synthesis Reaction Buffer (5X). First strand cDNA was synthesized using random hexamer primer and M-MuLV Reverse Transcriptase (RNase H^-^). Second strand cDNA synthesis was subsequently performed using DNA Polymerase I and RNase H. Remaining overhangs were converted into blunt ends via exonuclease/polymerase activities. After adenylation of 3’ends of DNA fragments, NEBNext Adaptor with hairpin loop structure were ligated to prepare for hybridization. In order to select cDNA fragments of preferentially 150–200 bp in length, the library fragments were purified with AMPure XP system (Beckman Coulter, Beverly, USA). Then 3 ul USER Enzyme (NEB, USA) was used with size-selected, adaptor-ligated cDNA at 37°C for 15 min followed by 5 min at 95°C before PCR. Then PCR was performed with Phusion High-Fidelity DNA polymerase, Universal PCR primers and Index (X) Primer. At last, PCR products were purified (AMPure XP system) and library quality was assessed on the Agilent Bioanalyzer 2100 system. The library preparations were sequenced on an Illumina Hiseq2500 platform and 150-bp paired-end reads were generated.

### Bioinformatics analysis

The raw reads were cleaned by removing adapter sequences, reads containing ploy-N and low quality reads through in-house perl scripts. At the same time, Q20, Q30, GC-content and sequence duplication level of the clean data were calculated. All the downstream analyses were based on clean data with high quality. The left files (read1 files) from all libraries/samples were pooled into one big left.fq file, and right files (read2 files) into one big right.fq file. Transcriptome assembly was accomplished based on the left.fq and right.fq using Trinity v2.2.0 [18] with min_kmer_cov set to 2 by default and all other parameters set default. Unigenes were annotated based on the following seven databases: Nr, Nt, Pfam[19], KOG/COG, Swiss-Prot, and KO and Gene Ontology (GO) [20], using BLAST with a cutoff E-value of < 10^−5^.

### Identification of simple sequence repeats (SSRs)

SSRs among all unigenes were identified using the MISA [21] (http://pgrc.ipk-gatersleben.de/misa/misa.html), and primer for each SSR was designed by Primer3 [22] (http://primer3.sourceforge.net/releases.php).

### Differential expression analyses

Gene expression levels were estimated by RSEM [23] for each sample by following steps: Clean data were mapped back onto the assembled transcriptome, and then Read count for each gene was obtained from the mapping results and normalized to reads per kb of exon model per million mapped reads (RPKM). For each sequenced library, the read counts were adjusted by edegR program package through one scaling normalized factor[24]. Differential expression analysis of three samples was performed using the DEGseq R package v1.21.1 [25]. P value was adjusted using q-value which was defined as the multiple testing analog of the p-value[26]. q-value<0.005 and |log2 (foldchange)|>1 was set as the threshold for significantly differential expression. Variations in gene expression levels were analyzed for specific comparisons that encompass two categories: (i) different developmental stages (AF and nymphs, AM and nymphs); (ii) sex differences (AF and AM). GO enrichment analysis of the DEGs was determined using the GOseqR packages[20]. Kyoto Encyclopedia of Genes and Genomes (KEGG)[27] pathway enrichment analysis was conducted using KOBAS software[28].

### Quantitative Real-time PCR

The samples collections and their total RNA isolation were same as described above. The cDNA was generated using PrimeScript RT Master Mix (Perfect Real Time) (TaKaRa). The qRT-PCR primers were designed using IDT Primer Quest (http://www.idtdna.com/primerquest/Home/Index) and shown in S4 Table. The qRT-PCR was performed on the ABI Prism^®^ 7500 (Applied Biosystems, Carlsbad, CA, USA) and the reaction was conducted in 20 μl reaction system containing 10 μl 2×GoTaq^®^qPCR Master Mix (Promega, Madison, WI, USA), 10 μM of each primer pair, 100ng cDNA and nuclease-free water. The following PCR-cycling condition was used for the qRT-PCR, 2 min at 95°C for 10 s, 40 cycles of 30 sec at 95°C and 1 min at 60°C. Relative RNA variations were normalized and corrected with the level of the *actin* gene (NCBI accession number KX108734). All experiments were performed in three biological replicates with three technical replicates. The relative expression quantities of each gene was calculated by the comparative2^-ΔΔCt^ method[29].

## Results

### Transcriptome sequencing and reads assembly

To obtain a comprehensive understanding of molecular mechanisms that control *C*. *Ciliata* biology at distinct life stages and sex difference, total RNA was extracted from nymphs, AF and AM samples, followed by sequencing with the Illumina Hiseq sequencing platform. The sequencing data were deposited in the NCBI Sequence Read Archive (SRA, http://www.ncbi.nlm.nih.gov/Traces/sra); BioProject ID number was PRJNA312006. The accession numbers were SRR3170921, SRR3170922 and SRR3170923, respectively. In total, 212,259,810 clean reads were assembled into a total of 83,754 transcripts from the nymphs, AF and AM libraries (Tables 1 and 2). The AF sample produced the most data (76,903,406 clean reads) and the AM sample produced the least amount of data (65,036,134 clean reads). The Q20 scores (the average quality value) were above 95% (Table 1). Moreover, the overall GC content of the clean reads was high similarities, ranging from 44.87% to 46.24%. The assembled reads from three samples representing the different life stages and sex difference generated a total length of 47,464,477 bp and 60,879 unigenes, with a mean contig length of 780bp and a N50 of 1,678 bp (Table 2). Analysis of the size distributions revealed that 11,459 unigenes were over 1,000 bp in length.

**Table 1:** Output statistics from AM, AF and Nymphs of *C*. *Ciliata*.

Samples	Total Raw Reads	Total Clean Reads	Clean bases(G)	Q20%	GC %
Nymphs	73,934,914	70,320,270	8.79	95.04	46.24
AM	67,466,456	65,036,134	8.13	95.16	45.93
AF	79,748,574	76,903,406	9.61	95.24	44.87
All	221,149,944	212,259,810	26.53		

**Table 2:** Assembly statistics from AM, AF and Nymphs of *C*. *Ciliata*.

	Total Number	Total Length (bp)	Mean Length (bp)	N50
Transcripts	83,754	86,443,735	1,032	2,319
Unigenes	60,879	47,464,477	780	1,678

### Function Annotation

All 60,879 *C*. *Ciliata* unigenes were subjected to the seven public databases (Table 3) for functional annotations. There were 26.81% of them (16,322 unigenes) matched against the Nr database, and 7.42% of them (4,519) had specific matches to nucleotide sequences in the GenBank Nt database, and 20.66% (12,581) were similar to proteins in the Swiss-Prot database (Table 3).

**Table 3:** Summary of functional annotations of *C*. *Ciliata* unigenes.

	Number of Unigenes	Percentage (%)
Annotated in Nr	16,322	26.81
Annotated in Nt	4,519	7.42
Annotated in KO	5,783	9.49
Annotated in SwissProt	12,581	20.66
Annotated in PFAM	14,246	23.4
Annotated in GO	14,349	23.56
Annotated in KOG	8,967	14.72
Annotated in all Databases	1,846	3.03
Annotated in at least one Database	20,579	33.8

**Fig 1:**
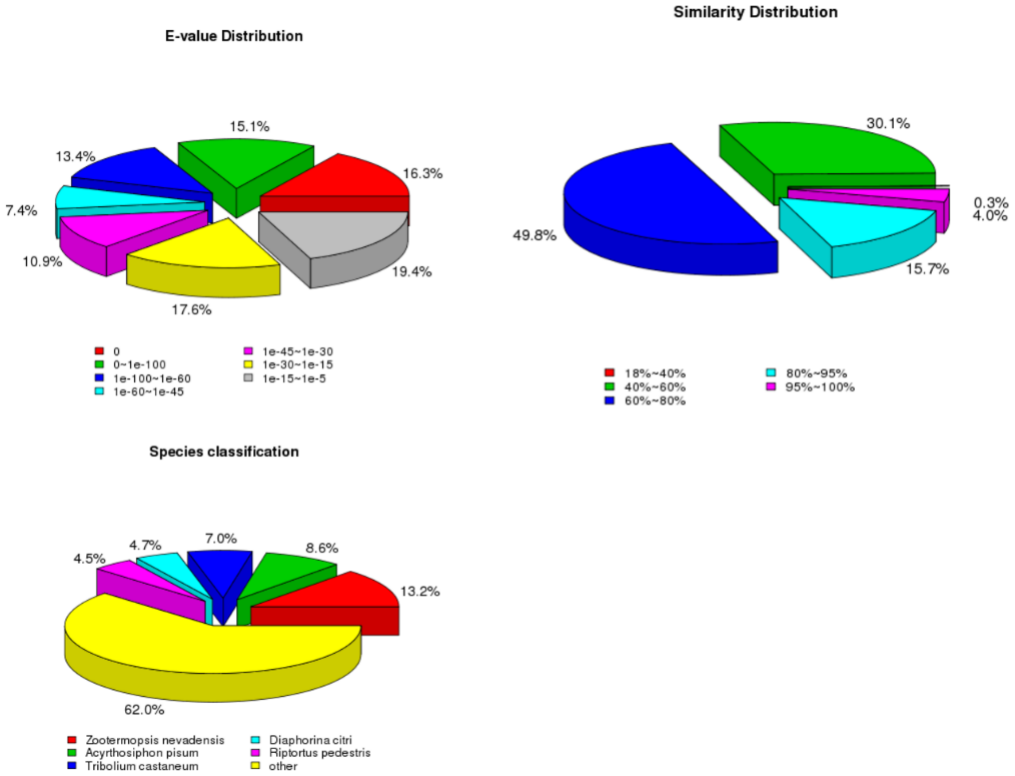
Pie charts showing distribution of the BLASTxmatches of *C*. *Ciliata* transcriptome unigenes against Nr databases. (a) E-values distribution, (b) similarity distribution, and (c) species classification.

Statistical tests on the distribution of the BLASTx[30] matches of *C*. *Ciliata* unigenes against Nr databases indicated that 63.0% of the annotated unigenes had significant homology (E-values<1e^−30^) to other sequences in Nr database, much higher than the percentage of homologous sequences with E-values between 1e^−5^ and 1e^−30^ (37%) (Fig 1a). The similarity distributions suggested that 69.5% sequences had a similarity more than 60%, while 30.4% sequences had a similarity ranging from 18% to 59% (Fig 1b). For main species distribution matched against Nr database, *C*. *Ciliata* unigenes have closely matched with sequences of the dampwood termite *Zootermopsis nevadensis* (13.2%), the pea aphid *Acyrthosiphon pisum* (8.6%), the red flour beetle *Tribolium castaneum* (7.0%), the Asian citrus psyllid *Diaphorina citri* (4.7%), and the bean bug *Riptortus pedestris* (4.5%) (Fig 1c).

**Fig 2:**
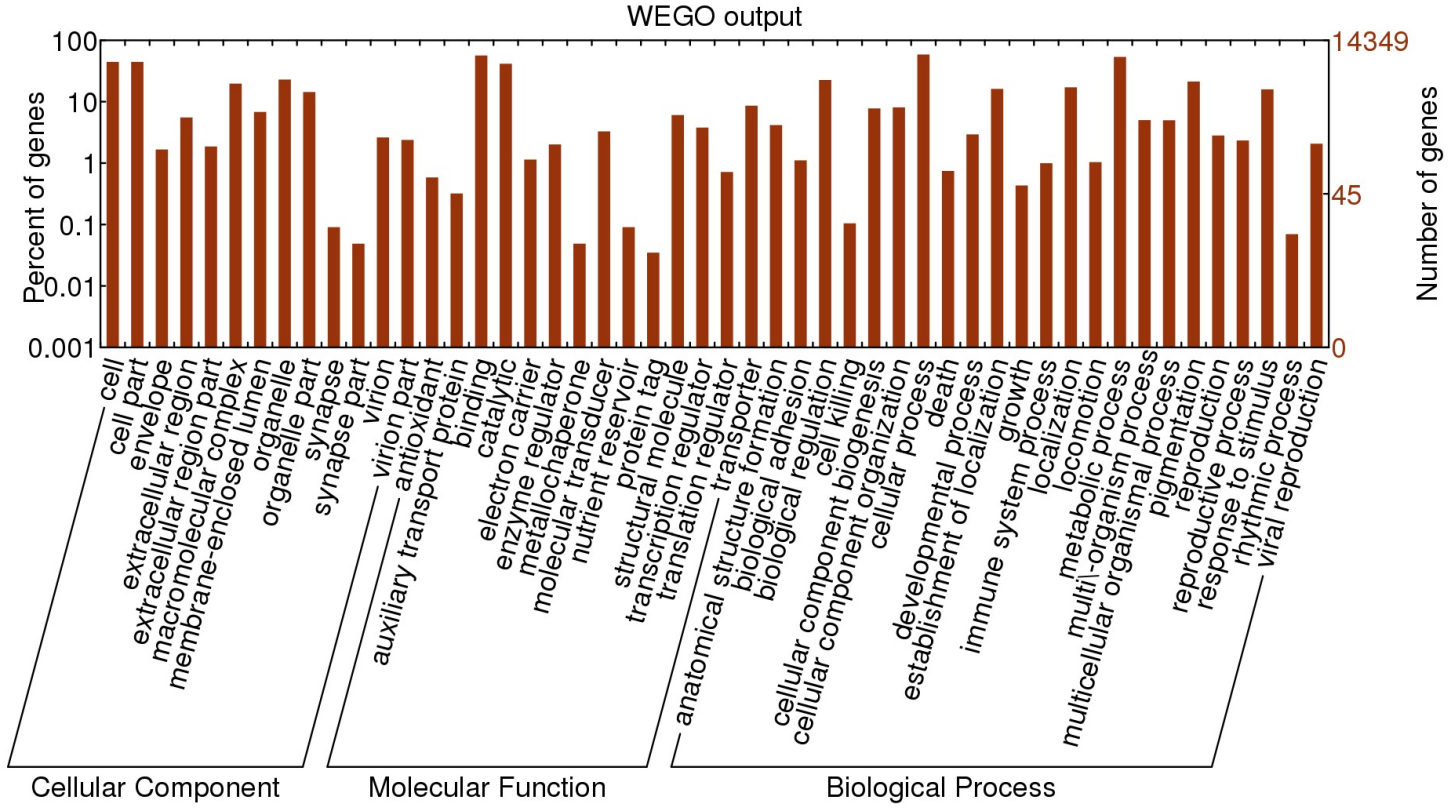
GO Classification of *C*. *Ciliata* unigenes according to the categories of Biological process, Molecular function and Cellular component.

The functional annotation of *C*. *Ciliata* unigenes by GO, KOG, and KEGG recovered diverse potential biological functions and processes. A total of 14,349 unigenes were annotated with GO functions, including 73.87% unigenes at the biological process level, 83.81% unigenes at the molecular function level, and 49.26% unigenes at the cellular component level. Within the Biological process GO category, Cellular process and metabolic process were most abundant. Cell and cell part terms were most abundant among the Cellular component category. For Molecular Function, unigenes were predominantly associated with binding and catalytic activity functions (Fig 2).

**Fig 3:**
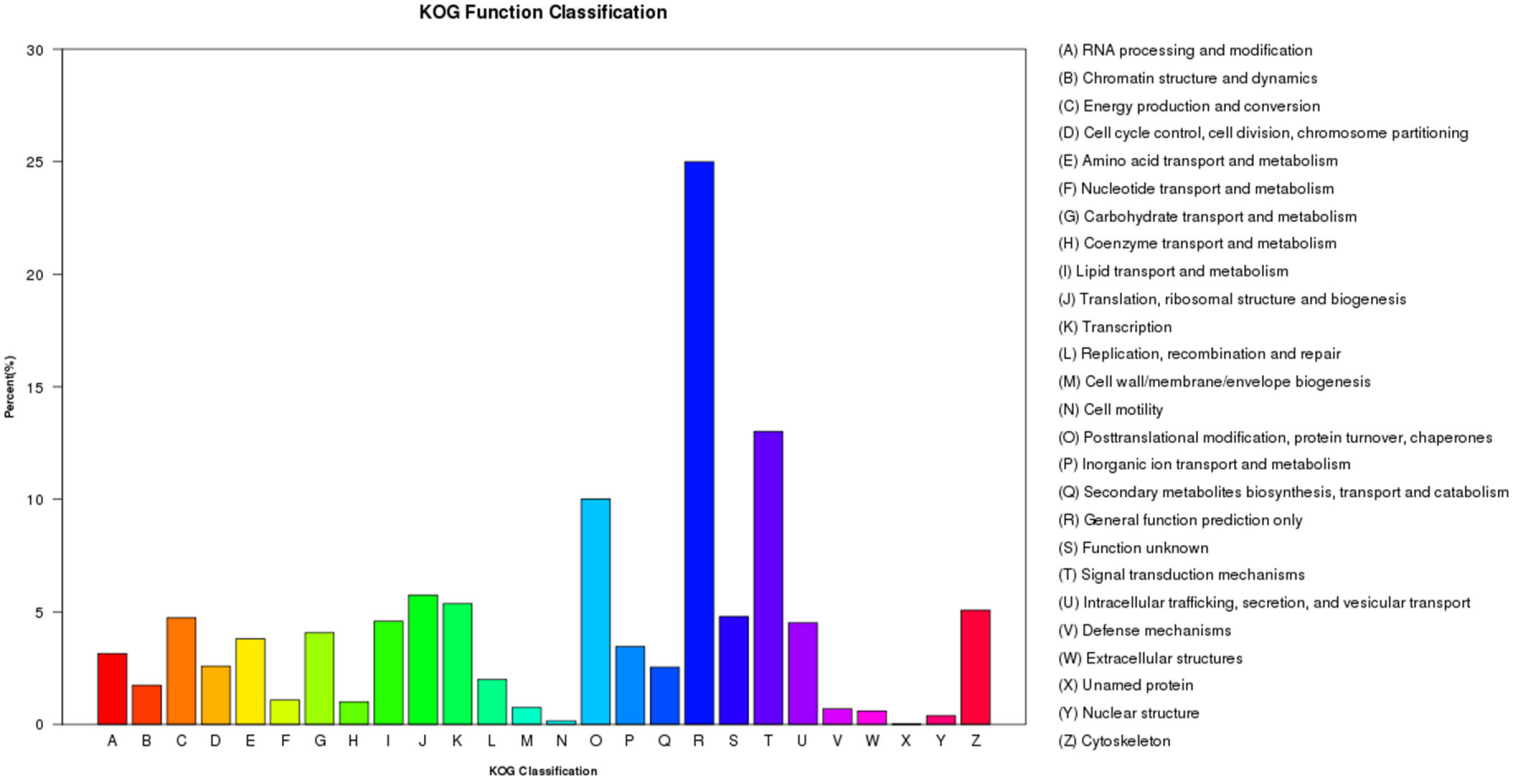
KOG annotations of *C*. *Ciliata* predicted proteins. A total of 8,967 predicted proteins have a KOG classification among the 26 categories.

After KOG-based annotation, a total of 8,967 annotated putative proteins were assigned to 26 KOG categories, mainly including general function prediction, signal transduction mechanisms, posttranslational modification, protein turnover, chaperones, translation, ribosomal structure and biogenesis, cytoskeleton, energy production and conversion, lipid transport and metabolism, and so on (Fig 3).

**Fig 4:**
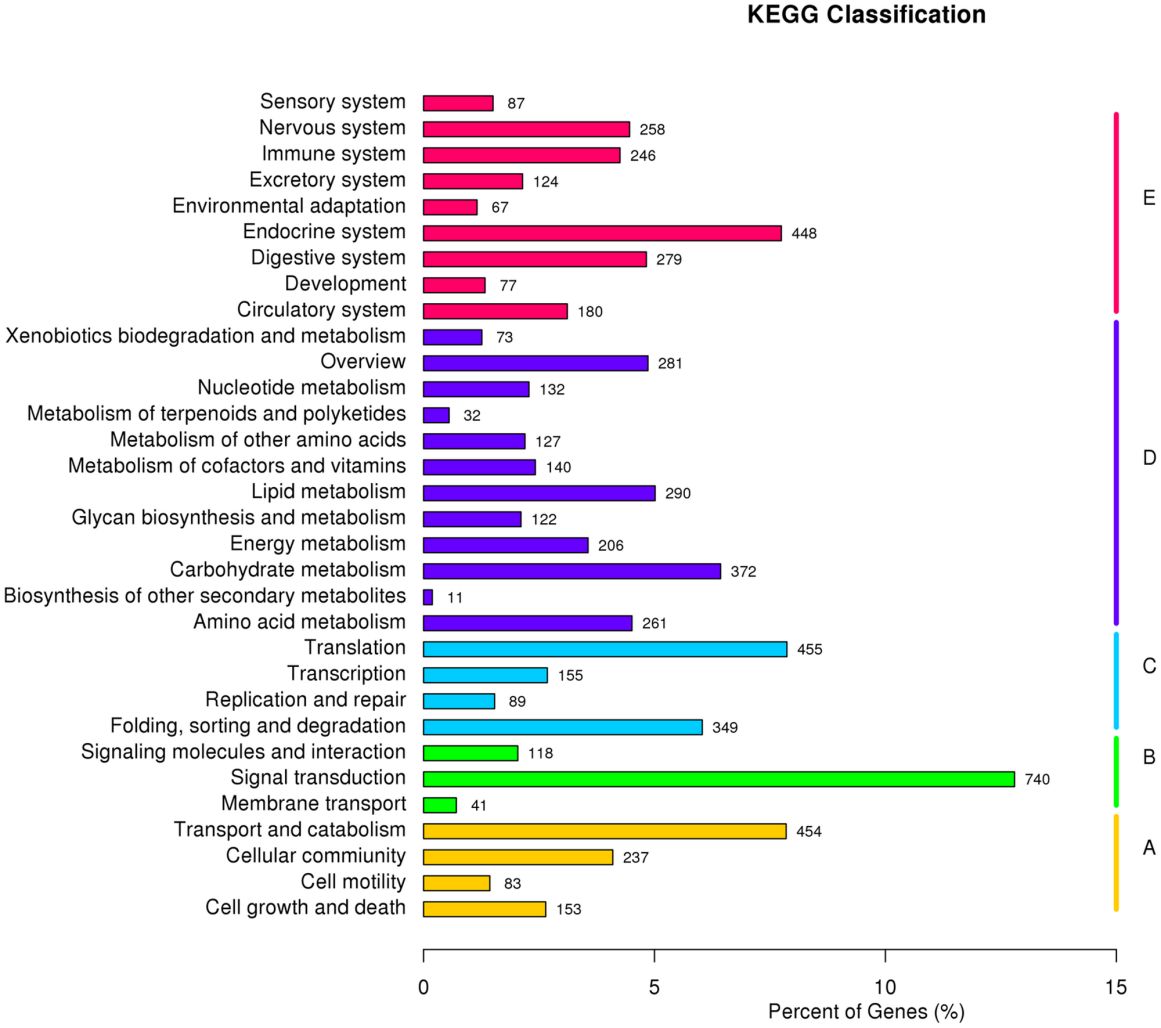
KEGG annotation of *C*. *Ciliata* predicted proteins.

We mapped the predicted proteins of the *C*. *Ciliata* to the reference authoritative pathways in KEGG for further functional categorization and annotation. In total, we assigned 5,783 proteins into 228 KEGG pathways, with 2,047 proteins (30.61%) being associated with metabolic pathways. The pathways involving the largest number of unigenes were signal transduction (740), followed by transport and catabolism (454), whereas biosynthesis of other secondary metabolism (11) was the smallest group (Fig 4).

### Protein coding sequence (CDs) prediction

**Fig 5:**
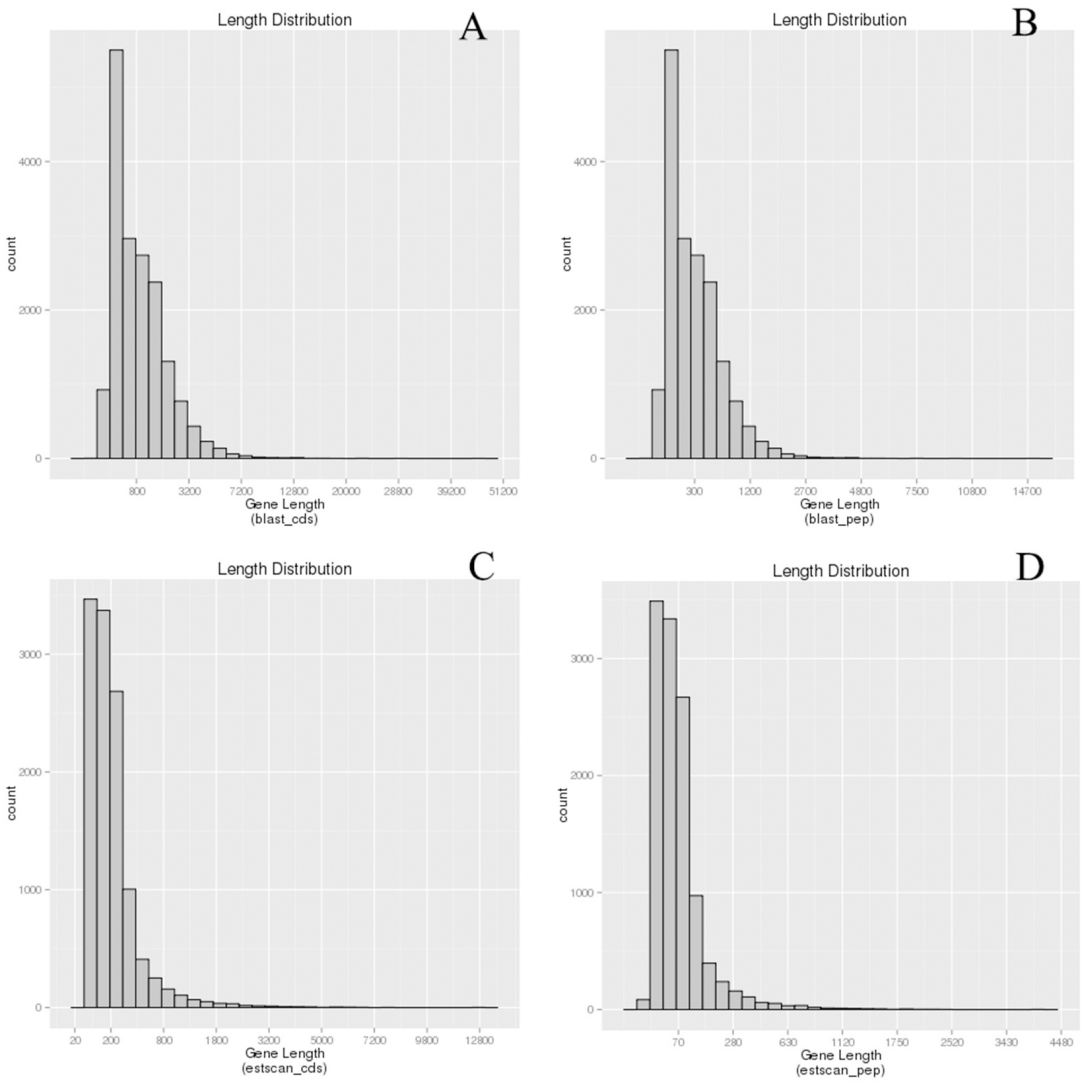
Coding sequence predictions of *C*. *Ciliata* transcriptome by BLASTx and ESTScan. (**a**) Length distribution of CDs using BLASTx (E-value < 10^−5^); (**b**) Length distribution of proteins using BLASTx; (**c**) Length distribution of CDs predicted by ESTScan, and **(d**) Length distribution of proteins using ESTScan.

All 60,879 unigenes were aligned against the protein databases, with the priority order of Nr, Swiss-Prot, KEGG and KOG. Using BLASTx, a total of 17,533 coding sequences (CDs) were obtained from unigenes sequences and translated into amino acid sequences (Fig 5A and 5B). Using the ESTScan[31], we identified 11,706 unigenes CDs that could not be matched to the above protein databases and translated them into amino acid sequences. Of these unigenes with CDs sequences, 4,187 were over 500 bp and 1,070 were over 1000 bp (Fig 5C and 5D).

### Frequency and distribution of SSRs

**Fig 6:**
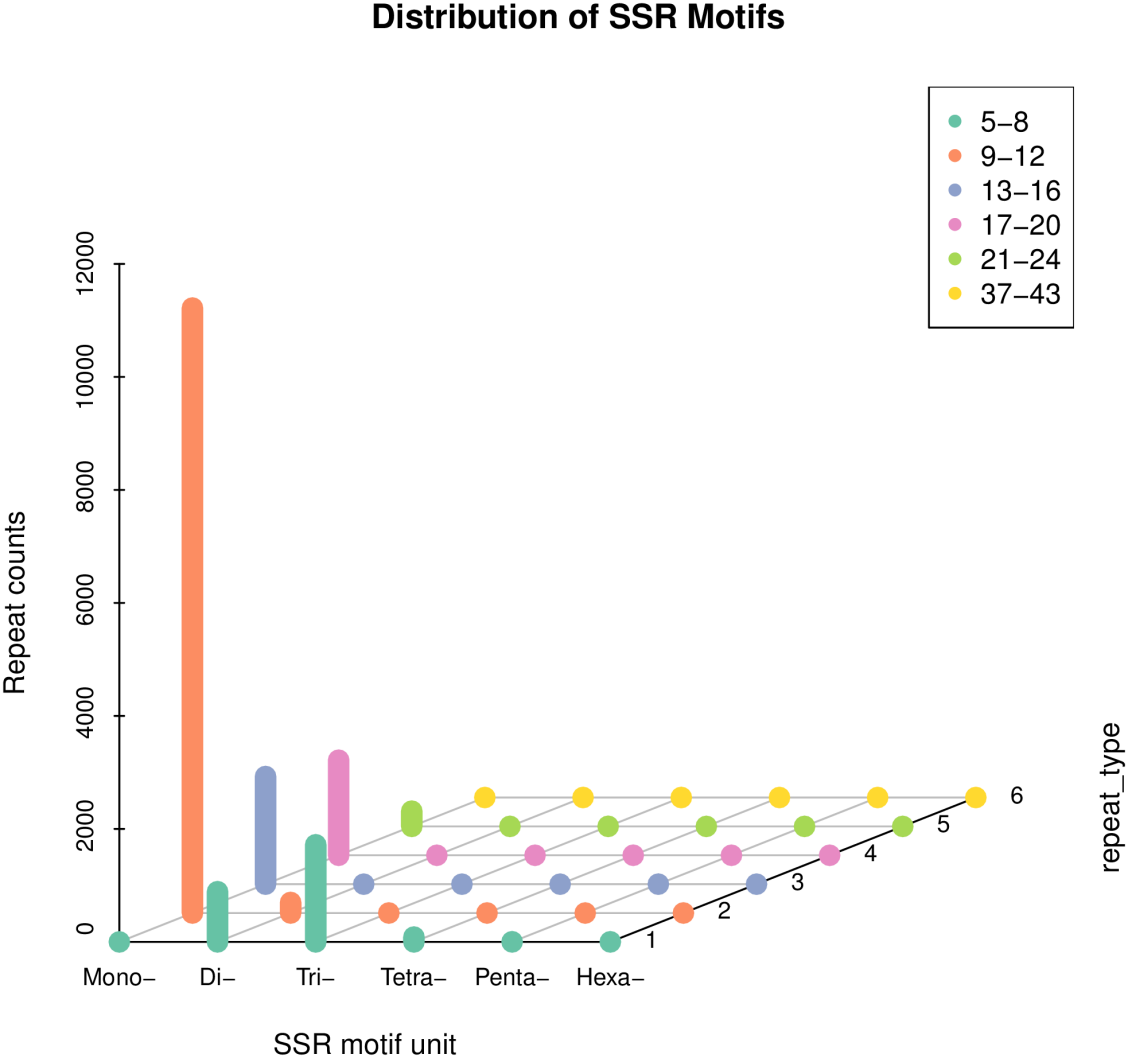
Frequency of SSRs in *C*. *Ciliata* transcriptomes.

We screened the *C*. *Ciliata* unigene dataset to mine potential SSRs for future population and evolutionary genetics analysis. Among 60,879 examined sequences (47,464,477bp), a total of 17,462 SSRs were identified and the number of SSRs containing sequences reached 12,105. A total of 9,300 pairs of potential SSR primers were designed. Furthermore, 3,359 sequences contained more than 1 SSRs. In the *C*. *Ciliata* transcriptome, the SSRs frequency was 28.68% and the distribution density was 0.37 per kb. Based on the repeat motifs, all SSRs loci were divided into 6 categories: mono-nucleotide repeats (14,566), di-nucleotide (1,078), tri-nucleotide (1,723), tetra-nucleotide (88), penta-nucleotide (4) and hexa-nucleotide repeats (3), respectively (Fig 6).

### Differential gene expression profile among *C*. *Ciliata* developmental stages and sex difference

**Fig 7:**
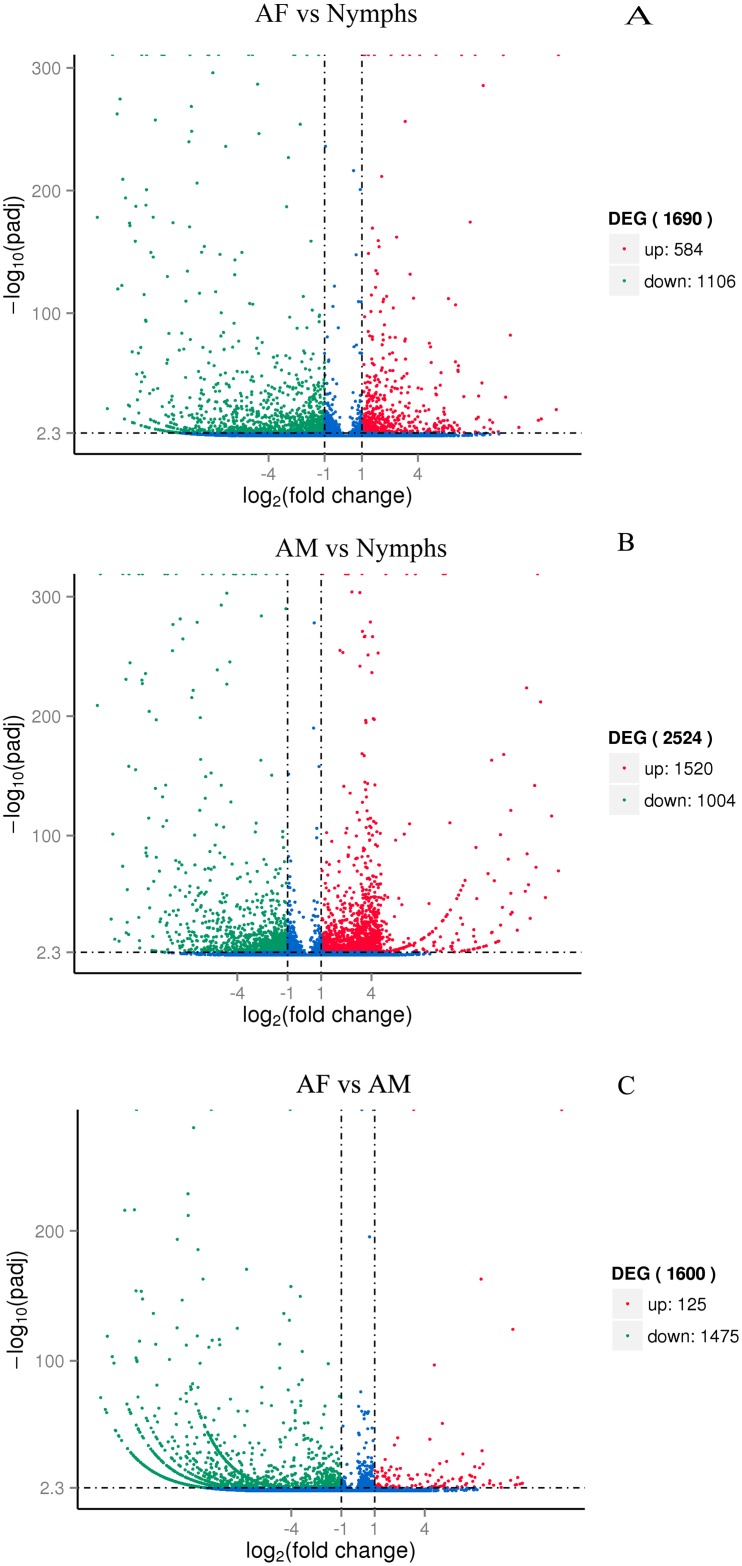
Volcano plots of differentially expressed unigenes. The abscissa represents the expressed levels fold change of unigenes in three different samples. The ordinate indicates the statistically significant difference degree. The lower and higher -log10 (p-adj) values mean greater differences. The scatters in diagram stand for each gene, the blue dot represents there was no significant difference of genes. The up-regulated and down-regulated genes were indicated by a red dot and green dot, respectively.

To specifically identify development-biased and sex-biased deferentially expressed genes, we calculated expression profiling to examine gene activity changes between selected developmental stages (nymph and adult male, nymph and adult female) and sexes difference (adult male and adult female) (Fig 7). DEGs (q-value <0.005 and |log2 (foldchange)| >1) were defined as genes that were significantly enriched or depleted in one sample relative to the other.

In total, 1,690 genes showed significant change in gene expression on comparison of AF and nymphs libraries, with 584 and 1,106 genes that were up-regulated and down-regulated in the AF library, respectively (Fig 7A). Among the top ten up-regulated DEGs, 5 showed defined functions, i.e., polyprotein (*Bemisia tabaci*), sodium-coupled monocarboxylate transporter 1-like (*Plutella xylostella*), group XV phospholipase A2-like (*Acyrthosiphon pisum*), chemosensory protein 8 (*Apolygus lucorum*), and cytochrome P450 6k1-like (*Microplitis demolitor*). In addition, among ten most down-regulated DEGs, five genes have predicted functions, i.e., hypothetical protein L798_05227 (Zootermopsis nevadensis), cuticle protein, putative (*Riptortus pedestris*), cuticle protein 19 (*Tribolium castaneum*), hypothetical protein TcasGA2_TC013819 (*Tribolium castaneum*), and hypothetical protein L798_05226, partial (*Zootermopsis nevadensis*) (S1 Table).

In the comparison between AM and nymphs, the expression profiles of 2,524 genes had changed. There were 1,520 and 1,004 unigenes that were significantly up-regulated and down-regulated in the AM library, respectively (Fig 7B). Among the top ten up-regulated genes, one displayed predicted functional gene in adult male library: the Cathepsin L1 in *Strongylocentrotus purpuratus*. The top ten down-regulated genes included defined functions: hypothetical protein L798_05227 (*Zootermopsis nevadensis*), uncharacterized protein LOC101741662 (*Bombyx mori*), Apolipoprotein D precursor, putative (*Pediculus humanus* corporis), hypothetical protein TcasGA2_TC013819 (*Tribolium castaneum*), cuticle protein 19 (*Tribolium castaneum*), and lysosomal aspartic protease (*Vollenhovia emeryi*) (S1 Table).

The comparison between AF and AM libraries revealed 1,600 genes were significantly difference, with 125 and 1,475 genes that were up-regulated and down-regulated in the AF library, respectively(Fig 7C). Five of the top ten up-regulated DEGs in the AF library have putative functions, i.e., polyprotein (*Bemisia tabaci*), protein bicaudal C homolog 1-B isoform X1 (*Cerapachys biroi*), ankyrin repeat protein (*Trichomonas vaginalis* G3), hypothetical protein captedraft_143611 (*Capitella teleta*) and cathepsin B (*Riptortus pedestris*). Among the top ten down-regulated DEGs, two genes have annotated functions, i.e., ornithine aminotransferase (*Riptortus pedestris*) and Cytosol aminopeptidase (*Pediculus humanus corporis*) (S1 Table).

### GO enrichment analysis for DGEs

DEGs sets in AF and nymphs libraries were investigated by GO enrichment analysis. For up-regulated DEGs in AF compared to nymphs, three terms oxidoreductase activity, catalytic activity, and cofactor binding were mostly highly enriched in the molecular function category. And the top three enriched terms in the cell components category were respiratory chain complex, respiratory chain and mitochondrion. For biological process, oxidation-reduction process, single-organism metabolic process, and monovalent inorganic cation transport were the top three dominant enriched terms. For the down-regulated DEGs in AF, chitin metabolic process, glucosamine-containing compound metabolic process and amino sugar metabolic process were the highest three biological process enriched. Only one term extracellular region was enriched in cellular component. The top three terms enriched in molecular function were structural constituent of cuticle, chitin binding, and structural molecule activity (S2 Table).

The GO enrichment analysis was conducted for the DEGs in AM sample, compared with nymphs (q-value < 0.05). The GO enrichment of up-regulated DEGs in AM was shown in S2 Table. In the molecular function category, the top three enriched terms were aminopeptidase activity, peptidase activity, acting on L-amino acid peptides and catalytic activity. In the cell components category, microtubule associated complex, microtubule cytoskeleton and cytoskeletal part were the top three dominant enriched terms. For biological process, proteolysis, tetraterpenoid metabolic process, and tetraterpenoid biosynthetic process were the mostly highly enriched. For the down-regulated DEGs in AM compared to nymph sample, chitin metabolic process, glucosamine-containing compound metabolic process and amino sugar metabolic process were the top-three biological process enriched by the down-regulated DEGs. Two terms extracellular region and extracellular matrix were the cellular component enriched by down-regulated DEGs. Four terms structural constituent of cuticle, chitin binding, structural molecule activity and serine-type endopeptidase activity were the enriched molecular function (S2 Table).

The GO enrichment analysis was conducted for the DEGs between AF and AM sample also (S2 Table). For the down-regulated DEGs in AF vs AM, serine-type endopeptidase activity, peptidase activity, acting on L-amino acid peptides, and serine-type peptidase activity were the top three enriched terms in the molecular function category. In the cell components category, microtubule cytoskeleton, microtubule associated complex and dynein complex were the top three dominant enriched terms. For biological process, proteolysis, protein metabolic process and tetraterpenoid metabolic process were the mostly highly enriched.

### Pathway enrichment analysis for DGEs

The KEGG pathway enrichment of the up-regulated DEGs in AF, compared with nymphs was investigated also. Seven pathways were enriched (q-value < 0.05) (S2 Table). For the down-regulated DEGs in the AF, five pathways including Glutathione metabolism, Amino sugar and nucleotide sugar metabolism, Arginine and proline metabolism, Gap junction, and Pathogenic *Escherichia coli* infection were enriched (S2 Table).

For the KEGG pathway enrichment of the up-regulated DEGs in AM, compared with nymphs. Two pathways Non-alcoholic fatty liver disease and Arginine and proline metabolism were enriched (q-value < 0.05). For the down-regulated DEGs in the AM, hippo signaling pathway—fly, pathways in cancer, basal cell carcinoma, amino sugar and nucleotide sugar metabolism and wnt signaling pathway were the enriched pathways (S2 Table).

However, no significant GO-term enrichment were observed for the up-regulated DEGs in AF. For KEGG pathway enrichment, the up-regulated DEGs in AF vs AM were enriched in the pathways lysosome and glycosphingolipid biosynthesis—globo series, according to KEGG pathway enrichment analysis. While, the down-regulated DEGs were not enriched significantly in AF.

### DEGs associated with metamorphosis, digestion and immune

To explore potential targets that might serve as novel pest management strategies for *C*. *Ciliata*, unigenes involved in metamorphosis, digestion and immunity were identified from the 1,291 DGEs between nymphs and adults (AM and AF)(S1 Fig). Based on the best BLAST matches against the Nr database, total of 10, 27 and 9 genes associated with metamorphosis, digestion and immunity were found, respectively (Table 4 and S3 Table). Among 10 metamorphosis-related unigenes, 6 were annotated respectively as methoprene-tolerant protein, broad, E7 series nuclear receptor, hormone receptors and ftz-f1. Other 4 unigenes belonged to juvenile hormone-related genes (S3 Table). In addition, we annotated 27 putative unigenes related to the digestive system (Table 4), which encoded 2 serine protease, 1 cysteine protease, 11 aminopeptidase, 6 carboxypeptidase, 5 lipase and 2 glucosidase. Among these 27 unigene, 12 were significantly differentially expressed between AF and AM. For 9 immunity-related unigenes, 4 were pathogen recognition genes encode pathogen surveillance proteins including 2 scavenger receptor and 2 Toll-like receptor. 4 unigenes were signaling pathway genes (Srpn or serpin). 1 unigene was effector gene encode antimicrobial peptide.

**Table 4:** Metamorphosis, digestion and immune related genes detected in the *C*. *Ciliata* DEGs dataset.

	Unigene ID		Fold Change	
		AF vs Nymphs	AM vs Nymphs	AF vs AM
**Metamorphosis-related genes**	**Met**			
	c37392_g1	**1.8916**	**1.1994**	0.48447
	**Broad**			
	c30286_g1	**-1.5684**	**-2.1682**	0.39217
	**E7 series nuclear receptor**			
	c34837_g1	**-4.2365**	**-4.2058**	-0.23834
	**Hormone receptors**			
	c38244_g1	**-3.5604**	**-3.9799**	0.21193
	**ftz-f1**			
	c32527_g1	**-1.144**	**-1.4728**	0.12109
	c38083_g2	**-1.1329**	**-1.4648**	0.12419
	**Others**			
	c36420_g1	**1.3299**	**1.1657**	-0.04342
	c23250_g1	**-3.1944**	**-3.3947**	-0.0072726
	c36792_g1	**-6.5889**	**-5.7741**	-1.0224
	c33463_g1	**1.3668**	**1.5055**	-0.34635
**digestion-related genes**	**Serine protease**			
	c39372_g1	**-3.0936**	**-4.0106**	0.70943
	c27683_g1	**-4.8423**	**-5.2471**	0.1971
	**Cysteine protease**			
	c36392_g1	**3.581**	**-5.9139**	**9.2873**
	**Aminopeptidase**			
	c36547_g2	**-5.1543**	**3.4303**	**-8.7922**
	c37887_g1	**-5.6824**	**-5.851**	-0.039035
	c24987_g1	**-6.1746**	**3.7993**	**-10.182**
	c26582_g1	**-11.509**	**3.3086**	**-15.025**
	c32511_g2	**-4.9939**	**3.1614**	**-8.152**
	c34521_g1	**-8.8125**	**3.3063**	**-12.326**
	c34676_g1	**-3.3304**	**3.1462**	**-6.6842**
	c34884_g1	**-9.6653**	**3.1381**	**-13.011**
	c35035_g1	**-1.6619**	**-2.1215**	0.25194
	c35539_g1	**-2.6068**	**-3.7812**	0.96677
	c36253_g1	**-5.6286**	**3.0675**	**-8.6928**
	**Carboxypeptidase**			
	c31658_g1	**-5.0132**	**-4.8101**	-0.41068
	c32354_g1	**-5.8394**	**-4.2195**	-1.8275
	c32694_g1	**-4.5936**	**-6.6929**	1.8917
	c34338_g2	**-4.4356**	**-5.7741**	1.1309
	c34575_g1	**1.9712**	**1.3423**	0.42127
	c35834_g1	**-1.3044**	**2.5209**	**-4.0329**
	**lipase**			
	c32378_g1	**-4.0463**	**-4.4767**	0.22277
	c14857_g1	**-2.4211**	**-2.5122**	-0.11657
	c27654_g1	**-5.8152**	**-5.0262**	-0.9966
	c28590_g1	**-3.1372**	**3.4538**	**-6.7987**
	c31359_g1	**-2.1681**	**-1.9684**	-0.40728
	**glucosidase**			
	c23488_g1	**-5.0055**	**2.6057**	**-7.608**
	c33442_g1	**1.9176**	**1.6349**	0.075112
**Immune-related genes**	**Scavenger receptor**			
	c34938_g1	**2.9408**	**3.0163**	-0.28313
	c30960_g1	**2.3447**	**1.956**	0.18107
	**Toll-like receptors**			
	c37763_g2	**-1.4859**	**-1.6096**	-0.083979
	c38863_g1	**-2.8667**	**-3.2647**	0.19034
	**Srpn or serpin**			
	c27683_g1	**-4.8423**	**-5.2471**	0.1971
	c34936_g1	**-3.1244**	**-2.9175**	-0.41454
	c39627_g1	**-5.8738**	**-2.4996**	-3.5818
	c39627_g2	**-5.3046**	**-2.6767**	-2.8355
	**Antimicrobial peptide**			
	c20205_g1	**7.85**	**8.1072**	-0.46479

Number in bold represents significantly difference: q-value <0.005 and |log2 (foldchange)| >1. The q-value and Nr annotations of these unigenes were in S3 Table.

### qRT-PCR analysis

To confirm the reliability of gene expression profiles in the sequencing data, 9 DEGs (difference between AF vs nymphs and AM vs nymphs) related to Juvenile hormone (c33463_g1, Juvenile hormone epoxide hydrolase 1; c37392_g1, methoprene-tolerant protein; c23250_g1, juvenile hormone binding protein-like precursor; c30286_g1, broad), cellular homeostasis (c20097_g1, small heat shock protein), defense (c20205_g1, defensin 4), molting and metamorphosis (c34837_g1, ecdysone-induced protein 78C), digestive (c31526_g1, salivary protein MYS2 precursor) and one cuticle protein (c36547_g6, Larval cuticle protein A3A) were chosen randomly and examined by the qRT-PCR in AF, AM and nymphs. In addition, 7 DEGs (difference between AF vs AM) related to sex difference (c37363_g1, vitellogenin receptor), zinc metabolism (c28326_g1, ADIPOR-like receptor) and other functions (c36825_g1, myosin-IIIb-like isoform X5; c38424_g1, protein bicaudal C homolog 1-B isoform X1; c33040_g1, ATP synthase subunits, mitochondrial isoform X1; c24503_g1, Atlastin; c36078_g1, Calpain-7) were examined in AF and AM.

**Fig 8:**
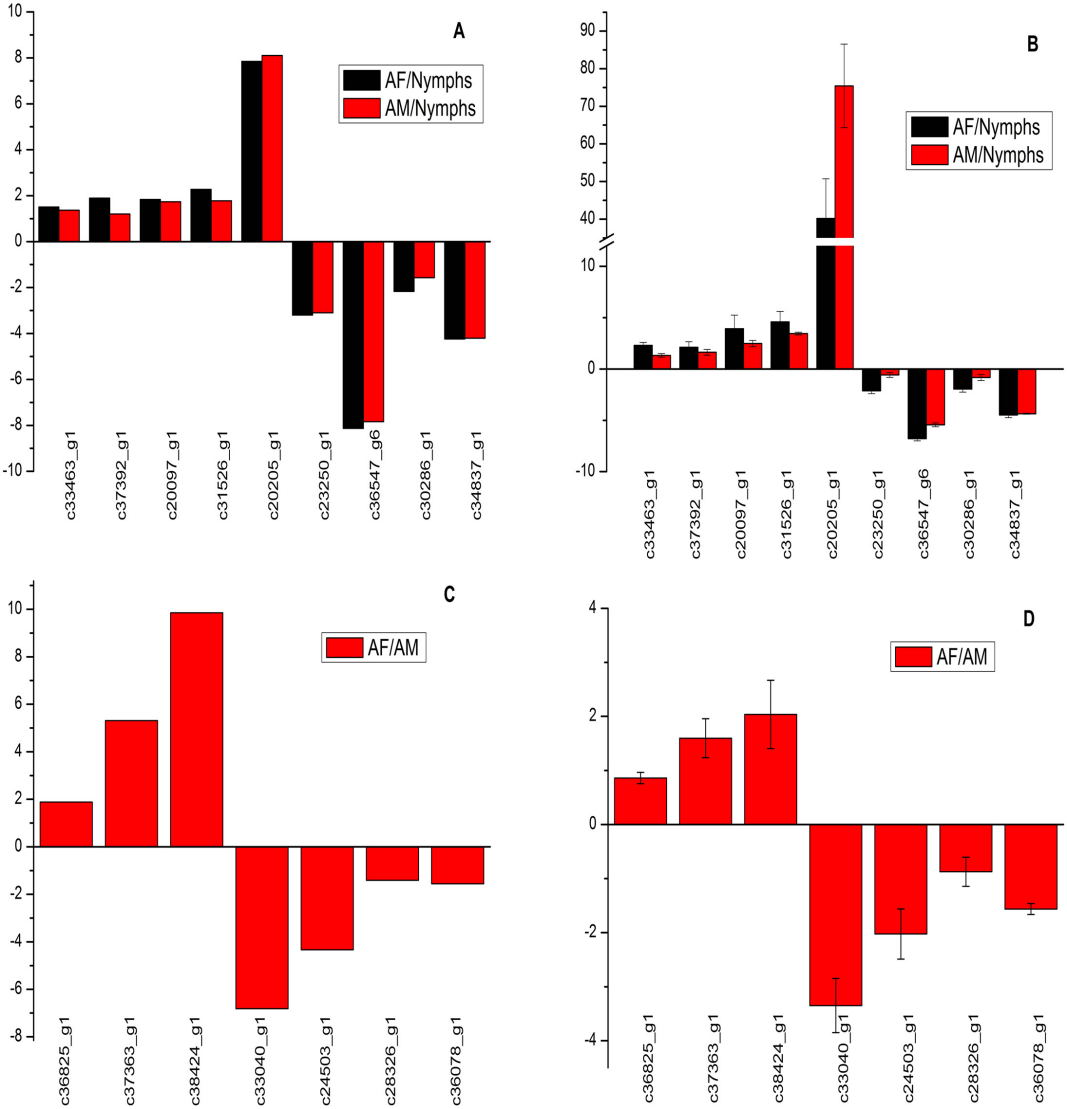
qRT-PCR validations results of DEGs in *C*. *Ciliata*. a. The fold changes for 9 DEGs (difference between AF vs nymphs and AM vs nymphs) from RPKM value in RNA-Seq data. b. The fold changes of these 9 DEGs in AF, AM and nymphs from qRT-PCR data. The fold changes of the 9 DEGs were calculated as the log2 value of each AF/nymph and AM/nymph comparison from RNA-Seq and qRT-PCR data. c. The fold changes for 7 DEGs (difference between AF vs AM) from RPKM value in RNA-Seq data. d. The fold changes of these 7 DEGs in AF and AM from qRT-PCR data. The fold changes of the 7 DEGs were calculated as the log2 value of each AF/AM comparison from RNA-Seq and qRT-PCR data. qRT-PCR were performed in triplicates. Error bars indicate the standard error of the mean. Significant difference was detected in all 9 DEGs between AF vs nymphs, AM vs nymphs (t-test, P<0.05) and all 7 DEGs between AF vs AM (t-test, P<0.05).

All the designed primers were listed in S4 Table. Results from the qRT-PCR experiment were demonstrated consistent expression patterns with the relative expression levels obtained by RNA-Seq data analyses and shown similar results in up- or down-regulation of *C*. *Ciliata* genes (Fig 8).

## Discussion

In this subject, the transcriptomes of *C*. *Ciliata* were sequenced by the Illumina platform. We identified a total of 60,879 unigenes from *C*. *Ciliata* transcriptome and only 26.81% showed specific NCBI Nr protein database matches (Table 3). This suggests that the transcriptome data provide abundant information besides the now available Nr protein sequences. Thus, our result make a significant contribution to the current molecular resources for this invasive forestry pest of plane trees (*Platanus* spp.), and provides a framework for understanding the development and sex difference. This dataset could serve as not only a valuable resource to better understanding of biology and physiology of *C*. *Ciliata*, but may also contribute to the development of novel pest manipulation strategies for *C*. *Ciliata*. Furthermore, this first large-scale transcriptomic dataset for *C*. *Ciliata* overall offers a valuable genetic resource for gene function and evolutionary research on Tingidae species.

Comparison of gene expression among the different developmental stages and different sex in the current experiment is helpful for identification of deferentially expressed genes across this pest's development and sexual dimorphism, thereby expanding our current knowledge of 60,879 *C*. *Ciliata* gene expression profiles. This result will contribute to future research on molecular feature of metamorphosis, digestion and immune, developmental mechanisms, and sex-determination mechanisms of this and other Tingidae species. During the developmental stage of *C*. *Ciliata*, the 60.22% of DEGs were found to be up-regulated and 65.48% were found to be down-regulated in the AM and AF when compared with the nymphs, respectively. In addition, a large number of genes showed developmental stage-related expression levels (e.g., juvenile hormone related genes, chemosensory protein, cuticular protein, etc) that were likely associated with *C*. *Ciliata* developmental process. Results From the comparison between AF and AM demonstrate that 125 and 1,475 unigenes were up-regulated and down-regulated in the AF, respectively. Vitellogenin receptor and protein bicaudal C were found to be up-regulated in AF vs AM, similar with previous study in Brown Planthopper *Nilaparvata lugens*[32]. Other various genes validated by qRT-PCR such as the Atlastin, ADIPOR-like receptor and the Calpain-7 gene appear to be sex-biased genes. In current study, A total of 19 juvenile hormone related genes were identified in our transcriptome data (S5 Table), the genes are more abundant than the previous study in another hemipteran pest *Diaphorina citri*, in which 13 juvenile hormone genes were found by transcriptome analysis[33]. The transcriptional expression levels of four juvenile hormone related genes (c33463_g1, Juvenile hormone epoxide hydrolase 1; c37392_g1, methoprene-tolerant protein; c23250_g1, juvenile hormone binding protein-like precursor; c30286_g1, broad) was further validate by qPCR in AF, AM and nymphs. Furthermore, many of sex biased genes (e.g. transformer protein, odorant binding protein, cuticular protein) identified from the whitefly *Bemisia tabaci* [34] and mirid bugs *Adelphocoris suturalis*[35] were also investigated in our transcriptome data, thereby further enriching our understanding of sex determination and differentiation in non-model insect pests.

In current study, many important DEGs in *C*. *Ciliata* transcriptome were detected which were potentially related to various key processes in the life of *C*. *Ciliata* (e.g., metamorphosis, digestion and immune, sex difference). These genes may offer novel molecular targets for developing new insecticides to control *C*. *Ciliata*. Further examination is necessary for characterizing these genes in development or sex differences in *C*. *Ciliata*, using a combination molecular biology, cell biology, gene knockout and knockdown approach (e.g., gene expression, electrophysiology analysis, morphological analysis, behavior analysis, CRSPR/Cas9[36] and RNAi [14]).

It is noteworthy that there is no annotation available for a large number of DEGs revealed in current study. These DEGs (536 unigenes between AM vs nymphs, 373 unigenes between AF vs nymphs, 391 unigenes between AF vs AM) showed significantly up-regulated or down-regulated patterns during development and sex difference. These new unigenes provides a unique resource for further studies of the developmental mechanism or sex determination mechanisms in *C*. *Ciliata*.

We identified 17,462 microsatellite DNA loci from the transcriptome dataset of *C*. *Ciliata*. In comparison to FIASCO methods for microsatellite isolation[37,38], transcriptomes provide an economical, fast, sensitive and high-throughput screening method. It also produces multiple types of microsatellite simultaneously. These microsatellite DNA loci found in our study will facilitate future research in population genetic structure, gene flow studies, and parentage analysis of *C*. *Ciliata*, as well as other Tingidae species[39,40].

## Supporting Information

S1 Fig Venn diagram of the DEGs detected in AF, AM and nymphs libraries. (TIF)

S1 Table Top ten DEGs in each library of comparisons. (XLS)

S2 Table GO and KEGG enrichment analysis for DEGs. (XLS)

S3 Table Metamorphosis, digestion and immune related genes detected in the *C*. *Ciliata* DEGs dataset. (XLS)

S4 Table Primers used in this study for qRT-PCR validation, including Nr annotation information for each unigenes. (XLS)

S5 Table Juvenile hormone related genes identified in the *C*. *Ciliata*. transcriptome. (XLS)
