# Supplementary figures and images for: Sequencing and Characterization of the Invasive Sycamore Lace Bug Corythucha ciliata (Hemiptera: Tingidae) Transcriptome
Source: PLoS One. 2016 Aug 5;11(8):e0160609. doi: 10.1371/journal.pone.0160609 (PMC4975459; doi:10.1371/journal.pone.0160609)

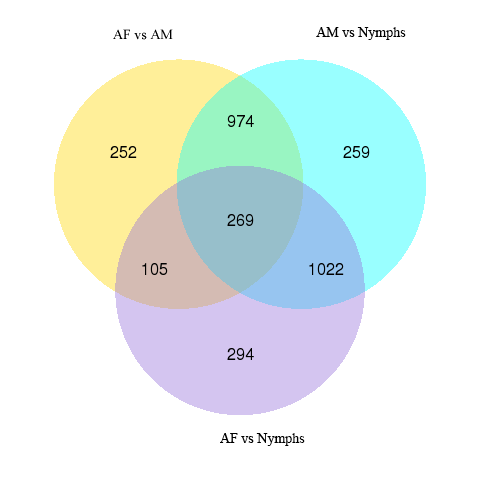

Supplement: S1 Fig — (TIF) [file pone.0160609.s001.tif]
